# Characterisation of Collagen Re-Modelling in Localised Prostate Cancer Using Second-Generation Harmonic Imaging and Transrectal Ultrasound Shear Wave Elastography

**DOI:** 10.3390/cancers13215553

**Published:** 2021-11-05

**Authors:** Wael Ageeli, Xinyu Zhang, Chidozie N. Ogbonnaya, Yuting Ling, Jennifer Wilson, Chunhui Li, Ghulam Nabi

**Affiliations:** 1 Division of Imaging Sciences and Technology, School of Medicine, University of Dundee, Ninewells Hospital, Dundee DD1 9SY, UK; w.ageeli@dundee.ac.uk (W.A.); c.z.ogbonnaya@dundee.ac.uk (C.N.O.); 2 Diagnostic Radiology Department, College of Applied Medical Sciences, Jazan University, Al Maarefah Rd, P.O. Box 114, Jazan 45142, Saudi Arabia; 3 Division of Population Health and Genomics, School of Medicine, University of Dundee, Dundee DD1 9SY, UK; 110023382xyz@gmail.com; 4 Imaging Science and Technology, School of Medicine, University of Dundee, Dundee DD1 9SY, UK; y.x.ling@dundee.ac.uk; 5 Department of Pathology, Ninewells Hospital, Dundee DD1 9SY, UK; jennifer.wilson7@nhs.scot; 6 School of Science and Engineering, University of Dundee, Dundee DD1 4HN, UK; c.li@dundee.ac.uk

**Keywords:** prostate, collagen, second-generation harmonics, shear wave, elastography, ultrasound

## Abstract

**Simple Summary:**

The study investigated the prostate cancer microenvironment and the correlation between peritumoral collagen and aggressivity of cancer. We used novel and advanced imaging including next-generation sequencing to characterise the expression of collagen in the extracellular matrix. Type and contents of collagen varied according to the Gleason grade in prostate cancer in men with localised prostate cancer undergoing radical surgery. The collagen contents orientation seen on second harmonic generation imaging varied with the grade of cancer and can predict clinically significant prostate cancer. Increased expression of Col1A1 is present in cancer tissues and associated with increased tissue modulus as measured by ultrasound shear wave elastography in prostate cancer. The study adds knowledge to our understanding of prostate cancer and defines the microenvironment of clinically significant prostate cancer, a disease more likely to impact survival and outcome of men who suffer from it.

**Abstract:**

Prostate cancer has a poor prognosis and high mortality rate due to metastases. Extracellular matrix (ECM) re-modelling and stroma composition have been linked to cancer progression, including key components of cell migration, tumour metastasis, and tissue modulus. Moreover, collagens are one of the most significant components of the extracellular matrix and have been ascribed to many aspects of neoplastic transformation. This study characterises collagen re-modelling around localised prostate cancer using the second harmonic generation of collagen (SHG), genotyping and ultrasound shear wave elastography (USWE) measured modulus in men with clinically localised prostate cancer. Tempo-sequence assay for gene expression of COL1A1 and COL3A1 was used to confirm the expression of collagen. Second-harmonic generation imaging and genotyping of ECM around prostate cancer showed changes in content, orientation, and type of collagen according to Gleason grades (cancer aggressivity), and this correlated with the tissue modulus measured by USWE in kilopascals. Furthermore, there were clear differences between collagen orientation and type around normal and cancer tissues.

## 1. Introduction

Prostate cancer is a public health issue with a significant impact on society because of its high incidence rate, and it is the second leading cause of cancer death in men around the world [1]. Prostate cancer development and progression to metastases is now widely recognised as a phenomenon of complex interactions between the cells of the prostate epithelial compartment and the surrounding stromal compartment in which they survive [2]. Cancer development normally starts with cells acquiring mutations that enable them to proliferate uncontrollably and overcome intrinsic mechanisms that regulate tissue homeostasis. Cells evolve over time to include the tumour microenvironment, consisting of various stromal cells, immune cells, and the extracellular matrix (ECM) in which all cells are embedded [3,4,5]. Several studies have reported that the biophysical and biochemical properties of the tumour microenvironment differ from those in normal tissues [6,7,8]. 

Several characteristics of the tumour microenvironment have been identified, including acidosis, high interstitial fluid pressure, hypoxia, and increased ECM modulus [2]. Liu et al. [9] summarised dynamic changes in the ECM due to cancer-associated fibroblasts (CAFs) as “desmoplasia” and suggested that these alterations were related to cancer metastases. Measurement of tissue modulus could potentially provide insights into the characteristics of the tumour microenvironment. According to Zhou et al. [9], the “stiff rim” sign was a unique characteristic of breast lesions on ultrasound shear wave elastography (SWE) imaging. However, the cause of biological characteristics of tissue modulus in prostate cancer is not well understood. CAFs secrete a high level of ECM proteins, including type I and type II collagen, fibronectin (Fn), and express oncofetal isoforms of Fn [8,10,11,12,13]. The alteration of matrix modulus is an immediate result of contractility-driven ECM re-modelling. Bundling collagen filaments and crosslinking collagen networks by CAFs cause additional modulus in the ECM [14,15]. Furthermore, collagen regulation is a crucial histopathological characteristic that distinguishes reactive stroma from the normal stroma and is linked to prostate cancer progression [16,17,18,19].

The elastic modulus of prostate cancer tissue is higher than that of normal prostate glandular tissue [19], and changes in elasticity can indicate an abnormal pathologic mechanism as tissue elasticity is a result of increased cell density and collagen formation [16,17]. Recently, there has been a surge of interest in measuring the elastic properties of prostate cancer [18,20,21,22]. 

Ultrasound shear wave elastography (USWE) is a promising imaging technique that measures tissue modulus and is increasingly applied for cancer detection [23]. Previous studies reported that USWE can distinguish malignant from benign in prostate cancer [24,25]. Previous studies have also reported that USWE based on its tissue modulus measurement has varying sensitivity, specificity, positive predictive value, and negative predictive value in the detection of prostate cancer [21,25,26], and these results may be linked to pathological features of cancer lesions such as fibrosis, hyperplasia, prostatitis, and ECM. What contributes to the increased modulus of cancers measured by USWE remains unknown and poorly understood. Furtherance of knowledge, particularly image characterisation of epithelial-stromal microenvironmental features would be a significant advantage in understanding the behaviour of prostate cancer, including its response to therapy. Collagen forms a major component of the tumour microenvironment and influences tumour behaviours through signalling pathways [27]. Changes in collagen content in the extracellular matrix have been ascribed to the mutual feedback loop that influences prognosis, recurrence, and resistance in cancers. 

Imaging and quantification of collagen deposition around cancer would be a significant advantage in understanding cancer and its progression. Second-harmonic generation of collagen (SHG) microscopy is a common imaging technique for visualising and characterising noncentrosymmetric 3D structures such as collagen [28]. We have previously reported its application in prostate cancer biopsy tissues [29]. In this study, we aim to:

Characterise collagen orientation and distribution around prostate cancer in comparison to normal tissue using SHG and correlate this with the Gleason grade of prostate cancer. 

## 2. Materials and Methods

### 2.1. Study Design and Patients

This was a prospective, protocol-driven study with ethical approval obtained through the East of Scotland Ethical committee and Caldicott permission (IGTCAL5626) to access healthcare follow-up data [30]. Tissue request approval was granted from the Tayside tissue bank, University of Dundee, Ninewells Hospital and Medical School, Dundee, UK. 

Inclusion criteria were: confirmed prostate cancer on TRUS guided biopsies, coupled with USWE imaging, and confirmed by radical prostatectomy. Patients were excluded by the following: previous radiotherapy or resection/intervention or hormonal treatment, including the use of 5 alpha-reductase inhibitors. 

Thirty men undergoing radical prostatectomy with three risk categories were recruited in this study: low-risk GS (GS = 6) prostate cancer (*n* = 10); intermediate-risk GS (GS 3 + 4) prostate cancer (*n* = 10); and high-risk GS (GS 8 or more) prostate cancer (*n* = 10). Patient-specific customised 3D moulds were printed using imaging and 3D printed according to our published protocol [31].

### 2.2. Ultrasound Shear Wave Elastography

All men had ultrasound shear wave elastography (USWE) before surgery. The USWE technique measures the shear wave speed produced using a specialised ultrasound transducer SE 12-3 that generates and measures shear wave speed A dynamic map of the tissue modulus (described as a Young Modulus) is reflected as a different speed in each tissue area in real-time [32,33]. All USWE images were taken with patients in either a lateral or lithotomy position utilising a transrectal endocavitary transducer (SuperSonic Imagine, Aix en Provence, France). For each prostate lobe, the USWE mode was used to capture elastograms of the prostate from the cranial to the caudal direction. In transverse planes with a spacing of 4 to 6 mm, the USWE pictures were obtained from base to apex. The most likely cancer-carrying planes were identified, and 3D pictures were created offline. When scanning suspected cancer areas with the transducer rotated in different directions, the anomalies were verified, and the dimensions were measured accurately. USWE examinations were performed by an experienced urologist with more than 10 years of experience in transrectal ultrasound. 

### 2.3. Second-Harmonic Generation (SHG) Imaging

The histopathology of each cancer lesion (from the histopathology slides) was marked and graded by an experienced uro-pathologist (JW). The tissues of the 30 patients were scanned by SHG to investigate collage distribution and orientation in different grades of prostate cancer along with genotyping for collagen. The ROI was 0.5 cm in width and 2 cm in length. SHG imaging was carried out on an upright Leica SP8 multi-photon microscope system at Dundee Imaging Facility. An HC Plan Fluotar 10 × 0.3 NA objective lens was used for all imaging. The second harmonic signal was generated using 880 nm from a Spectra-Physics InSight laser, and a transmitted light image was generated using a 488 nm laser. Both the SHG and transmitted light images were collected onto separate PMTs in the forward (transmitted) position using frame sequential acquisition. The SHG signal was collected with a 440/20 nm bandpass filter and the transmitted light image was collected through a 483/32 nm bandpass filter. The Navigator function of the Leica LAS X software was used to define the region of the section for imaging and set focus points across that region. The frame size was set to 1024 × 1024 pixels, giving 1.082-micron pixels and a bit depth of 12. A bi-directional scan at 400 Hz was used for all acquisitions. Any phase inaccuracies of the bi-directional scan were corrected post-acquisition.

Collagen fibre alignment in each section was measured using Fourier transform second harmonic generation (FT-SHG), as mentioned in a previous study [29]. The processing algorithms were developed in MATLAB R2017a (The MathWorks, Natick, MA, USA) to quantify the alignment of collagen fibres in each section utilising Fourier transform second harmonic generation adapted from the previously reported studies [34,35,36,37,38,39,40,41]. This technique was used to identify the quantitative parameters representing collagen orientation and distribution in the radical prostatectomy of prostate cancer by assessing the spatial frequencies within an image using a 2D-FT. Fast Fourier transformations were used to make the 2D-FT images. It was then radially integrated over various angles, yielding a plot of amplitude as a function of orientation angle. The plot was then fitted with Gaussian fits. The orientation of fibres was depicted in the centre of the fit, and the width of the fit was a measurement of the randomness with which the fibres were distributed. The angular power spectrum is used to determine whether a region is isotropic or anisotropic. According to the experimental results and the literature [38], a single sharp peak in the angular power spectrum refers to a region containing fibres all oriented in the same direction, whereas multiple peaks in the power spectrum correspond to several various directions that fibres are oriented along. As a result, the A:I ratio measures the number of areas with strongly aligned fibres in contrast to those with randomly oriented fibres. The preferred orientation in each plane was defined as the direction along which the majority of fibres appeared to align. The high amplitudes in 2D-FT were on average aligned perpendicularly to the preferred orientation. The original image was divided into 100 sub-images with a size of 50 μm and classified into three groups: negligible, anisotropic, or isotropic. This was performed to rapidly calculate the preferred orientation. If the SHG signal intensity in the region was low or almost dark, it was considered negligible. The sub-images with preferential collagen fibre orientation were labelled anisotropic, while those with several different directions were labelled isotropic. The A:I ratio was used to determine the overall orientation of the collagen fibres in each region (the ratio of the number of anisotropic to isotropic sub-images).

### 2.4. Radical Prostatectomy Histopathology as Reference Standard

According to our published protocol, a unique customised 3D mould was designed for each patient in this study using imaging data and a 3D printer [31]. The moulds were printed prior to surgery based on the T2-weighted mpMRI prostate images. They were designed to keep prostates after surgical removal in the same form and orientation as seen on the MRI. The 3D mould has a series of evenly spaced parallel slits, each of which corresponds to a recognised slice of T2-weighted MRI. This allows pathologists to gross specimens in the same orientation as imaging.

### 2.5. Genomic Analysis of mRNA Expression

We used TempO-Seq assays (BioSpyder., Inc. BIOCLAVIS, LTD, Biotechnology company in Glasgow, Scotland), a highly efficient multiplexed molecular profiling technique to target sequencing-based RNA expression analysis on FFPE tissue sections of the human prostate. Our samples were fixed in a slide with two regions of interest mapped out from each sample (normal prostate tissue and cancerous tissue) before excision of 10 mm was taken for both regions. The samples were analysed using the BioClavis’ internal process controls and with the Human Whole Transcriptome v2.0 panel with standard attenuators. In TempO-Seq, each Detector Oligo contains a sequence complementary to an mRNA target and a universal for all of the targeted gene primer binding sites. They anneal directly in juxtaposition to each other on the targeted RNA template in a way they can be ligated together. Subsequently, the Ligated detector oligos were PCR-amplified using a primer set (single-plex PCR reaction, with a single primer pair for each of the samples), which introduced both the adaptors needed for sequencing and a sample-specific barcode. Then the barcode sequences flanked by the target sequence were properly introduced into the standard Illumina adaptors to permit standard dual-index sequencing of the barcodes and deconvolution of sample-specific reads from the sequencing data using a standard Illumina software. All the amplified and PCR and barcoded samples were linked into a single library for sequencing and the sequencing reads were de-multiplexed using a standard sequencing instrument software for each sample. Subsequently, we used the barcodes to create individual FASTQ files. A data matrix of gene expression level with sample names as column headers and gene names as rows was used to identify COL1A1 and COL3A1 used for further analysis.

### 2.6. Data Analysis

The following data were collected: Patient’s age (in years); prostate-specific antigen (PSA); prostate volume (in mL); prostate-specific antigen density (PSAD); modulus measurement using USWE (in kpa); Gleason Score (GS); and orientation of collagen fibres (A:I ratio) in the prostate cancer and benign areas. PSAD was calculated using PSA divided by the prostate volume. Continuous data were first tested to see if they were normally distributed by the Kolmogorov–Smirnov Test of Normality. The mean (m) and standard deviation (SD) were described if the variable followed a normal distribution. The median (M) and interquartile range (IQR) were presented if the variable did not follow a normal distribution. Independent-sample t-tests were used to compare the means of the continuous variables that were normally distributed. Categorical variables were reported as frequencies and proportions. In this study, a GS ≥ 4 + 3 was considered to be significant prostate cancer following the UCL 2 definition. Collagen orientation (A:I ratio) in normal and cancer prostate tissue were compared. Cancer tissue collagen orientation (A:I ratio) against different GS were compared as well. The relationship between prostate cancer tissue modulus and collagen orientation was examined by Pearson Correlation. The relationship between prostate cancer tissue collagen orientation and GS was examined by the Spearman rank correlation. Statistical analyses were conducted using SPSS V23.0. A gene expression analysis for identifying the collagen types was conducted to validate the results from SHG.

Data are available on request from the authors.

## 3. Results

**Figure 1:**
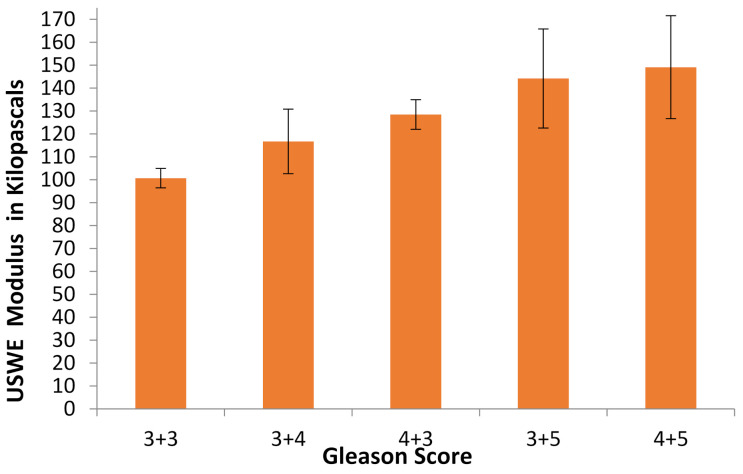
Bar chart of the Gleason score and USWE modulus in 30 patients with prostate cancer (the bar is the mean modulus in each Gleason score category with standard deviation sitting on the top of the bar), (r_s_ = 0.67, *p* < 0.001, *n* = 30).

A Spearman rank correlation was conducted to examine the relationship between the prostate cancer Gleason score and USWE modulus. The mean for collagen orientation in each Gleason score category was 100.7 ± 4.2, 116.7 ± 14.1, 128.5 ± 6.5, 144.2 ± 21.6 and 149.1 ± 22.5 (Figure 1). The relationship was positive, medium in strength and statistically significant (r_s_ = 0.67, *p* < 0.001, df = 28). 

**Figure 2:**
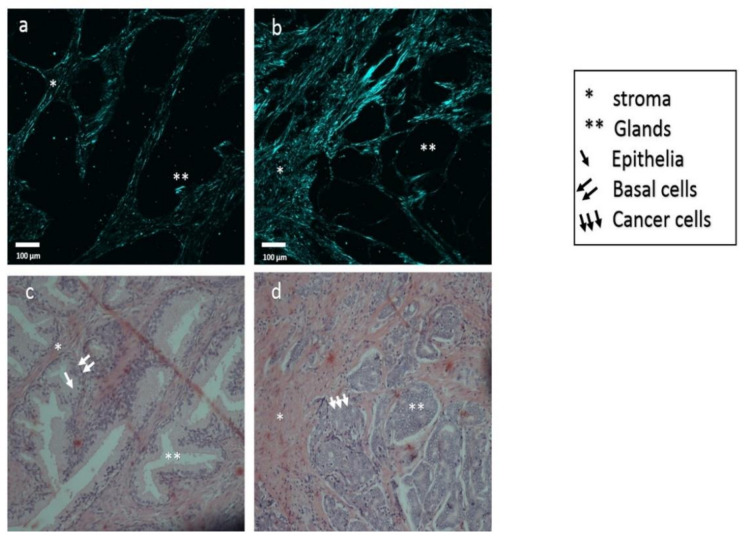
Comparison between normal prostate tissue and cancer prostate tissue. SHG images of collagen alignment in (**a**) normal prostate tissue and (**b**) cancer prostate tissue. Histopathology images of (**c**) normal prostate glands and (**d**) fused prostate glands occupied by cancer cells. The scale bar is 100 μm. The microscope magnification is 200×.

Figure 2 shows that the normal prostate tissue is composed of the gland and surrounding stroma. On the one hand, in Figure 2a, the typical gland is composed of two layers of cells (the inner layer is epithelia and the outer layer is basal cells) and has a papillary projection view. On the other hand, Figure 2d shows cancer prostate tissue that has lost the basement membrane and shows a disrupted epithelial layer compared to normal prostate tissue.

**Figure 3:**
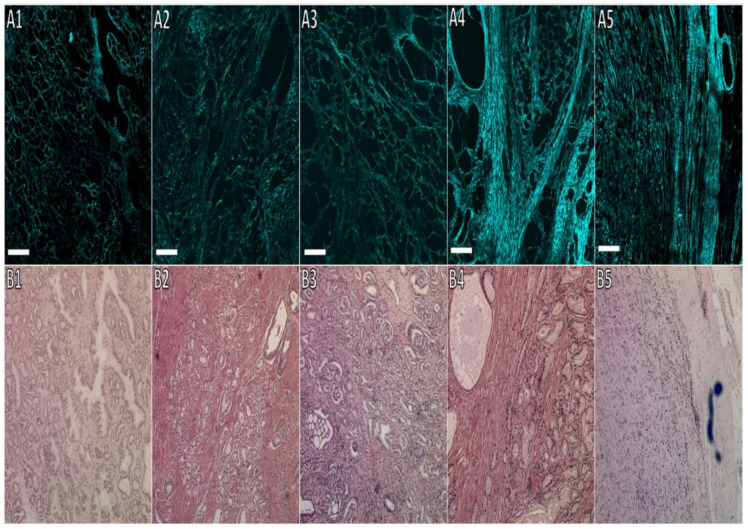
Prostate cancer sections with different Gleason scores. (**A1**–**A5**) SHG images, and (**B1**–**B5**) H&E images. In specimens with prostate cancer GS 3 + 3 (1), the SHG signals in prostate cancer were surrounding the glands with more closed margins; however, these became fainter and partially lost in GS 3 + 4 (2) and 4 + 3 (3). As the cancer is more aggressive GS (3 + 5 (4) and 4 + 5 (5)), the cancer cells fill the gland, and some spread to the stroma. In GS 4 + 5, the glands are fused, and there are only sheets of cancer cells left with no glandular shape. The scale bar is 300 μm, and the microscope magnification is 100×.

Furthermore, the shape of the gland in cancer tissue has changed from papillary to reticular. As expected, the SHG detected changes in the fibromuscular stroma containing the collagen. Compact, loose, crosslink, and more oriented collagen were observed in the prostate cancer tissue compared to normal prostate tissue. Increased GS of prostate cancer leads to increased collagen orientation and crosslinking, which may cause increasing prostate cancer tissue modulus. Figure 3 shows different grades of cancers and collagen orientation around cancer foci. 

The pattern of the SHG signal in the images can be distinguished between different GS, as seen in Figure 3. The A:I ratio was used to calculate the regularity of collagen fibre orientation and to compare GS across radical prostatectomies. In general, prostate cancer tissues collagens were found to be more aligned than prostate normal tissues. When comparing the collagen orientation in cancer and normal prostate tissue, after testing for normality, there was a significant difference in cancer A:I ratio (m = 3.21, SD = 1.09) and normal A:I ratio (m = 2.09, SD = 0.45); the F-test for the significance of the difference between the variances of the two samples was statistically significant (F = 5.94, *p* < 0.001), suggesting to use the t-test result assuming unequal sample variances; t(38.5) = 5.19, *p* < 0.001. These results suggest that collagen orientation in cancer prostate tissue is statistically higher than that in normal prostate tissue, as presented in Table 1. 

**Figure 4:**
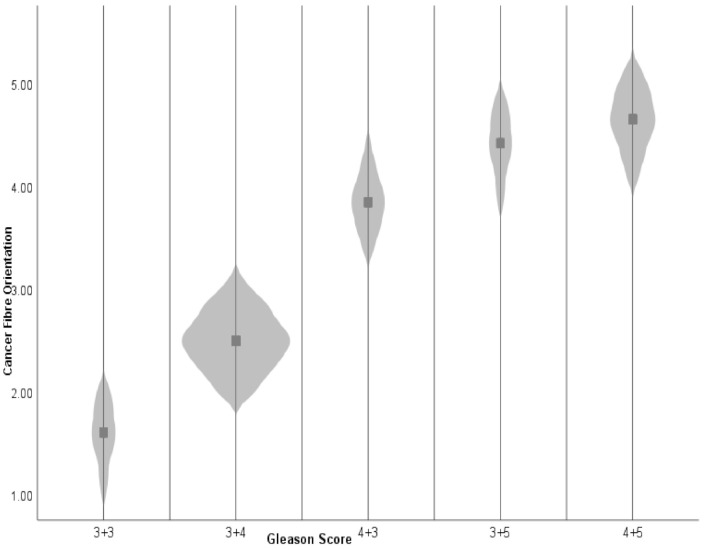
Violin Plot showing data distribution between cancer fibre orientation and Gleason score groups (grey rectangular sport in each violin is the mean of each group).

Furthermore, the A:I ratio increased with a higher GS, indicating that the collagen fibre became more oriented as the prostate cancer became more aggressive, as shown in Table 2 and Figure 4. 

When comparing collagen orientation in cancer and normal prostate tissue, after testing for normality, there was a significant difference in cancer A:I ratio in significant cancer (m = 4.57, SD = 0.26) and insignificant cancer (m = 2.62, SD = 0.72); F-test for the significance of the difference between the variances of the two samples was statistically significant (F = 7.91, *p* = 0.003), suggesting using the t-test result assuming unequal sample variances; t (27.72) = 10.87, *p* < 0.001. The results suggest that the cancer tissue collage orientation in significant prostate cancer patients is statistically higher than that in insignificant prostate cancer patients, as presented in Table 3.

**Figure 5:**
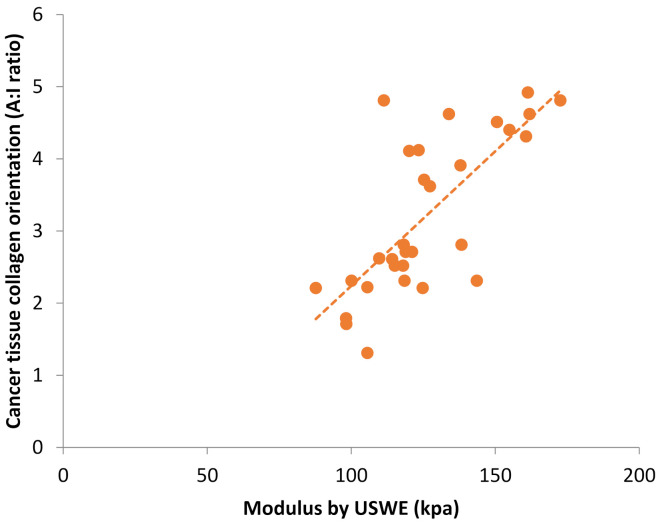
Scatter plot of the USWE modulus and SHG collagen orientation in 30 patients with prostate cancer (r = 0.74, *p* < 0.001, *n* = 30).

A Pearson correlation examined the relationship between prostate cancer modulus and tissue collagen orientation. The mean for modulus was 125.9 (SD = 21.6), and the mean for collagen orientation was 3.2 (SD = 1.1). The relationship was positive, strong in strength and statistically significant (r(28) = 0.74, *p* < 0.001), as seen in Figure 5.

**Figure 6:**
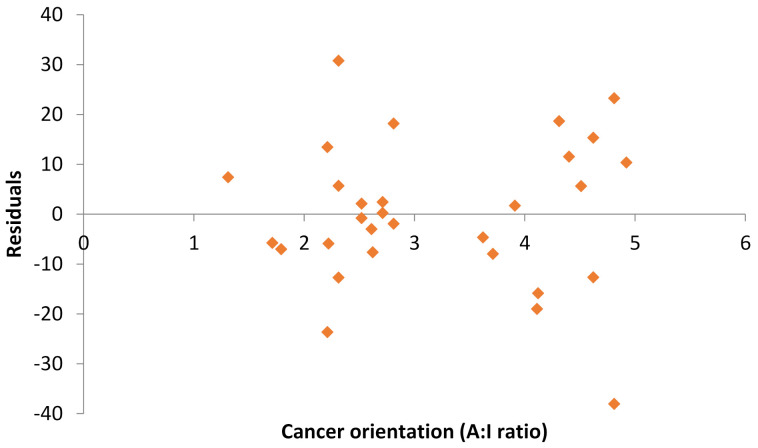
Residual plot between residuals and cancer orientation.

In order to measure how well the line fits the data, residuals were calculated and plotted against the explanatory variable, which is the A:I ratio (Figure 6). The plot showed no systematic curvature nor major outliers, which indicated it was a reasonable residual plot and therefore the line fits the data well.

**Figure 7:**
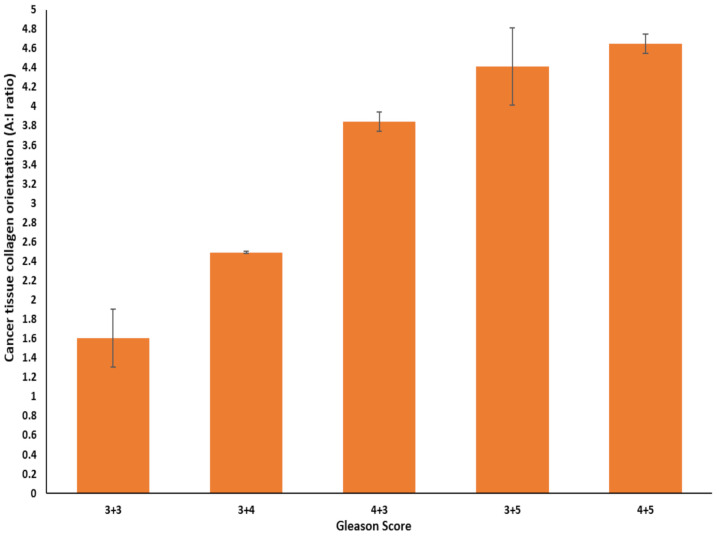
Bar chart of Gleason score and cancer collagen orientation in 30 patients with prostate cancer (the bar is the mean A:I ratio in each Gleason score category with standard deviation sitting on the top of the bar), r_s_ = 0.93, *p* < 0.001, *n* = 30.

A Spearman rank correlation was conducted to examine the relationship between prostate cancer GS and tissue collagen orientation. The mean for collagen orientation in each GS category was 1.6 ± 0.3, 2.5 ± 0.01, 3.8 ± 0.1, 4.4 ± 0.4, and 4.6 ± 0.1. The relationship was positive, very strong in strength and statistically significant (Spearman rank correlation coefficient r_s_ = 0.93, *p* < 0.001, df = 28), as shown in Figure 7.

The gene expression for COl1A1 in the cancer area was higher than Col3A1. The means of the gene expression level of COL1A1 in prostate cancer tissue in GS 3 + 3, 3 + 4, 4 + 3, 3 + 5, and 4 + 5 were 383, 920.6, 2729, and 927, respectively, while in COL3A1 they were 48.6, 114.2, 198.5, and 163.7, respectively. Col1A1 collagen demonstrates most of the fibres in cancer tissue compared to Col3A1 collagen.

## 4. Discussion

In this study, we investigated the prostate cancer modulus using USWE and further characterised this with SHG to detect the content and orientation of collagen. To validate the results, we presented gene expression to identify the collagen types associated with cancer modulus. Thirty patients with prostate cancer were recruited in this study. Using the MATLAB code on R2017a (The MathWorks, Natick, MA, USA) to identify the orientation of collagen in SHG images, the methodology appeared to offer a highly accurate and promising way of differentiating cancer tissue from normal tissue in the prostate gland. Moreover, it provided a precise characterisation of the GS based on the measurements of tissue modulus and orientation of collagen in cancer lesions. The study provides a mechanistic proof of tissue modulus by clearly showing type 1 collagen around cancer tissue with a high degree of preferred orientation. This increases with the grade of cancer. The dynamic behaviour of collagen around cancer provides tensile strength to tissue and may be responsible for tissue modulus similar to the observation by Whelan A et al. [42]. In fact, the longevity of prosthetic heart valves and their failure depends on the collagen orientation of the original structure [43], indirectly indicating that it is not the content of collagen but the orientation that contributes to the tensile strength of the tissues.

To address the limitations of existing prostate cancer detection techniques, a comprehensive strategy is required. The ability of USWE to significantly improve prostate cancer detection has been demonstrated. USWE identifies and locates tumours by detecting specific differences in tissue elasticity [44]. In the detection of prostate cancer, USWE has shown high sensitivity, specificity, and accuracy [20,22,25,26,45,46]. USWE provides elastograms that offer additional detail and clarity based on the measurement of tissue modulus. The range of tissue modulus could predict a higher GS cancer with the final histopathological examination of a radical prostatectomy specimen. In this study, we further characterised the modulus using the SHG optically nonlinear coherent process where the emitted light has exactly half the wavelength of the two-incident photon [47]. The technique specifically measures collagen fibres due to their crystalline and noncentrosymmetric properties. There were clear distinctions between cancer and normal lesions in the structural images of the prostate tissue of the radical prostatectomy in SHG images. Our observations are similar to other colleagues reporting tumour tissues had few and straight collagen fibres that displayed higher anisotropy values than normal skin and breast tissue [48]. When comparing breast cancer tissue to normal tissue, changes in collagen alignment have been observed in the stroma [49,50]. Raghu et al. reported that the ratio of collagen orientation in breast biopsy tissue was 2.8 ± 1.5 for normal samples and 11.6 ± 6.7 for cancer tissues [38]. Yuting et al. reported that the ratio of collagen orientation in prostate biopsy tissue was 1.34 ± 0.19 for normal samples and 2.82 ± 0.62 for cancer tissues [29]. However, in this study, the radical prostatectomies histopathology of a larger patient cohort included a bigger and more accurate area of tissue scanned using SHG signal obtained in the forward direction. Subsequently, the collagen pattern was then generated for each lesion, and the resultant collagen orientation was computed by FFT-SHG. A papillary collagen pattern was found in normal tissue, while a reticular and sparse collagen pattern was found in the cancer tissue. In normal prostate tissue, isotropic collagen fibres were observed without a preferred orientation. However, collagen orientation changed in prostate cancer tissue, and this became more obvious as the prostate cancer became more aggressive. In normal tissue, collagen fibres were oriented in different directions, whereas in cancers, a higher orientation (anisotropy) was observed. In this study, we investigated the relationship between the orientation of collagen and GS, as well as the relationship between collagen orientation and tissue modulus. The collagen orientation information addressed the increase in tissue modulus in the prostate cancer tissue compared to normal, benign prostate tissue. From the gene expression results, we observed that the amount of COL1A1 increased in cancer tissue compared to COL3A1. Tord et al. reported that COL1A1 collagen demonstrates most of the fibres in cancer tissue, which is similar to our results [48]. Liu et al. recently reported the potential role of COL1A1 in the development of metastases and suggested that this could be a target for future drug development in breast cancer [51].

## 5. Conclusions

The study using novel imaging provides mechanistic proof of tissue modulus, including orientation, dispersion and type of collagen around normal and prostate cancer tissues. There is a correlation between the type of collagen and the content of collagen, including orientation and prostate cancer aggressivity. Differences in collagen content, orientation, distribution, structures, and type play a critical role in prostate cancer modulus and thus act as a critical marker for distinguishing the different GS of prostate cancer from normal tissue.

## Figures and Tables

**Table 1:** Comparison of collagen orientation (A:I ratio) between normal and cancerous prostate tissue.

Tissue	*n*	df *	t-Value	*p*	Mean	∆mean	95% CI of ∆mean
Cancer Tissue	30	38.5	5.19	<0.001	3.21	1.12	0.68 to 1.56
Normal Tissue	30				2.09		

* df, degrees of freedom; ∆mean, the difference between the means in each group; F-test found unequal sample variances.

**Table 2:** The comparison of cancer orientation among prostate cancer and Gleason Grade.

Gleason Grade	Number of Patients (*n* = 30)	Cancer Orientation A:I Ratio (mean ± sd)	Minimum Cancer Orientation	Maximum Cancer Orientation
3 + 3	3	1.6 ± 0.28	1.31	1.79
3 + 4	14	2.5 ± 0.01	2.21	2.81
4 + 3	4	3.8 ± 0.1	3.62	4.12
3 + 5	3	4.4 ± 0.4	4.11	4.62
4 + 5	6	4.6 ± 0.25	4.31	4.92

**Table 3:** Comparison of cancer collagen orientation (A:I ratio) between clinically significant and insignificant prostate cancer.

Prostate Cancer	*n*	df *	t-Value	*p*	Mean	∆mean	95% CI of ∆mean
Significant Cancer	9	27.72	10.87	<0.001	4.57	1.95	1.58 to 2.32
Insignificant Cancer	21				2.62		

* df, degrees of freedom; ∆mean, the difference between the means in each group; F-test found unequal sample variances; Gleason score ≥ 4 + 3 was considered to be significant prostate cancer.

## Data Availability

The data are available for scrutiny from external requests.
